# ACADM inhibits AMPK activation to modulate PEDV-induced lipophagy and β-oxidation for impairing viral replication

**DOI:** 10.1016/j.jbc.2024.107549

**Published:** 2024-07-11

**Authors:** Quanqiong Wang, Qi Zhang, Xiaojie Shi, Naling Yang, Yanxia Zhang, Shifan Li, Yina Zhao, Shuxia Zhang, Xingang Xu

**Affiliations:** College of Veterinary Medicine, Northwest A&F University, Yangling, Shaanxi, China

**Keywords:** PEDV, β-oxidation, ACADM, lipophagy, AMPK

## Abstract

Porcine epidemic diarrhea virus (PEDV) belongs to the *Alphacoronavirus* genus within the *Coronavirus* family, causing severe watery diarrhea in piglets and resulting in significant economic losses. Medium-chain acyl-CoA dehydrogenase (ACADM) is an enzyme participating in lipid metabolism associated with metabolic diseases and pathogen infections. Nonetheless, the precise role of ACADM in regulating PEDV replication remains uncertain. In this study, we identified ACADM as the host binding partner of NSP4 *via* immunoprecipitation-mass spectrometry analysis. The interaction between ACADM and NSP4 was subsequently corroborated through coimmunoprecipitation and laser confocal microscopy. Following this, a notable upsurge in ACADM expression was observed during PEDV infection. ACADM overexpression effectively inhibited virus replication, whereas ACADM knockdown facilitated virus replication, suggesting ACADM has negative regulation effect on PEDV infection. Furthermore, we demonstrated fatty acid β-oxidation affected PEDV replication for the first time, inhibition of fatty acid β-oxidation reduced PEDV replication. ACADM decreased PEDV-induced β-oxidation to suppress PEDV replication. Mechanistically, ACADM reduced cellular free fatty acid levels and subsequent β-oxidation by hindering AMPK-mediated lipophagy. In summary, our results reveal that ACADM plays a negative regulatory role in PEDV replication by regulating lipid metabolism. The present study introduces a novel approach for the prevention and control of PEDV infection.

Porcine epidemic diarrhea virus (PEDV) is the pathogen that causes porcine epidemic diarrhea (PED); it is mainly infects and replicates within villous epithelial cells of the small intestine of pig. This infection leads to clinical manifestations such as watery diarrhea, vomiting, dehydration, and high mortality rates among neonatal piglets, resulting in substantial economic losses (1). PEDV is an enveloped, single-stranded positive-sense RNA virus with a 28 kb genome, classified within the *Alphacoronavirus* genus in the family Coronaviridae. Its genome contain seven ORFs encoding four structural proteins (containing nu-cleocapsid, N; envelope, E; membrane, M; spike, S), 16 nonstructural proteins (NSPs) and an accessory protein (ORF3) (2, 3). Virus protein is the carrier to realize its function, the NSP of PEDV plays an important role in the process of virus invasion into host cells, gene replication, transcription, translation and immune escape, exploring the interaction between NSPs and the host will help us better understand the pathogenesis of PEDV (4). PEDV NSP4 can upregulate the expression of proinflammatory cytokines and chemokines *in vitro* (5), but the mechanism of interaction between PEDV NSP4 and host is not yet fully understood.

Most viral infections typically trigger broad metabolic changes within host cells. Metabolic pathways such as lipid synthesis, β-oxidation, and glycolysis are often exploited by viruses to enhance replication and evade host immunity (6, 7). Lipids serve essential physiological functions in the body, including membrane synthesis and energy provision, which are crucial for virus proliferation (8). For example, dengue virus (DENV) infection can remodel lipid metabolism. During early infection, lipid droplets (LDs) are reabsorbed into the endoplasmic reticulum, providing lipids for endoplasmic reticulum expansion and replication complex formation (9). Later in infection, DENV induces lipophagy, mobilizing lipids from LDs for β-oxidation and energy generation (10, 11). Conversely, hepatitis C virus (HCV) reduces lipid β-oxidation to change cell energy by downregulating the expression of mitochondrial trifunctional protein, which increasing availability of lipids (12). However, the mechanism by which PEDV utilizes fatty acid β-oxidation is still unclear.

ACADM is lipid metabolism enzyme that catalyzes the first dehydrogenation step of β-oxidation; it is related to the breakdown of fatty acids in mitochondria and energy production (13, 14, 15). Medium-chain acyl-CoA dehydrogenase deficiency, arising from ACADM gene deletion or mutation, is a prevalent genetic metabolic disorder. Individuals with medium-chain acyl-CoA dehydrogenase deficiency may experience a variety of complications during fasting or stress, including drowsiness, vomiting, hypoketotic hypoglycemia, and biochemical abnormalities. In severe cases, it can lead to encephalopathy, seizures, coma, and fatality, highlighting ACADM's substantial role in metabolic diseases (16, 17). However, the involvement and mechanism of ACADM in PEDV infection have not been reported.

Here, we screened host NSP4-interacting proteins by immunoprecipitation-mass spectrometry to better understand the pathogenesis of PEDV and found that NSP4 can interact with ACADM. After PEDV infection, the expression of ACADM increased. In addition, our findings demonstrated that PEDV infection had the ability to induce fatty acid β-oxidation. However, when ACADM was overexpressed, it resulted in a decrease in PEDV-induced β-oxidation, which consequently impaired PEDV replication. Further research demonstrated that overexpression of ACADM had no effect on the expression of adipose triglyceride lipase, a key lipolysis enzyme. However, it inhibited PEDV-induced lipophagy to reduce the level of free fatty acids (FFAs), thereby inhibiting β-oxidation. At the same time, we confirmed that ACADM suppressed lipophagy by inhibiting AMPK. These studies demonstrate the distinctive role of ACADM in regulating lipid metabolism to damage PEDV replication and could be used to develop novel therapeutic strategies against PEDV infection.

## Results

### PEDV NSP4 interacts with the cellular protein ACADM

**Figure 1:**
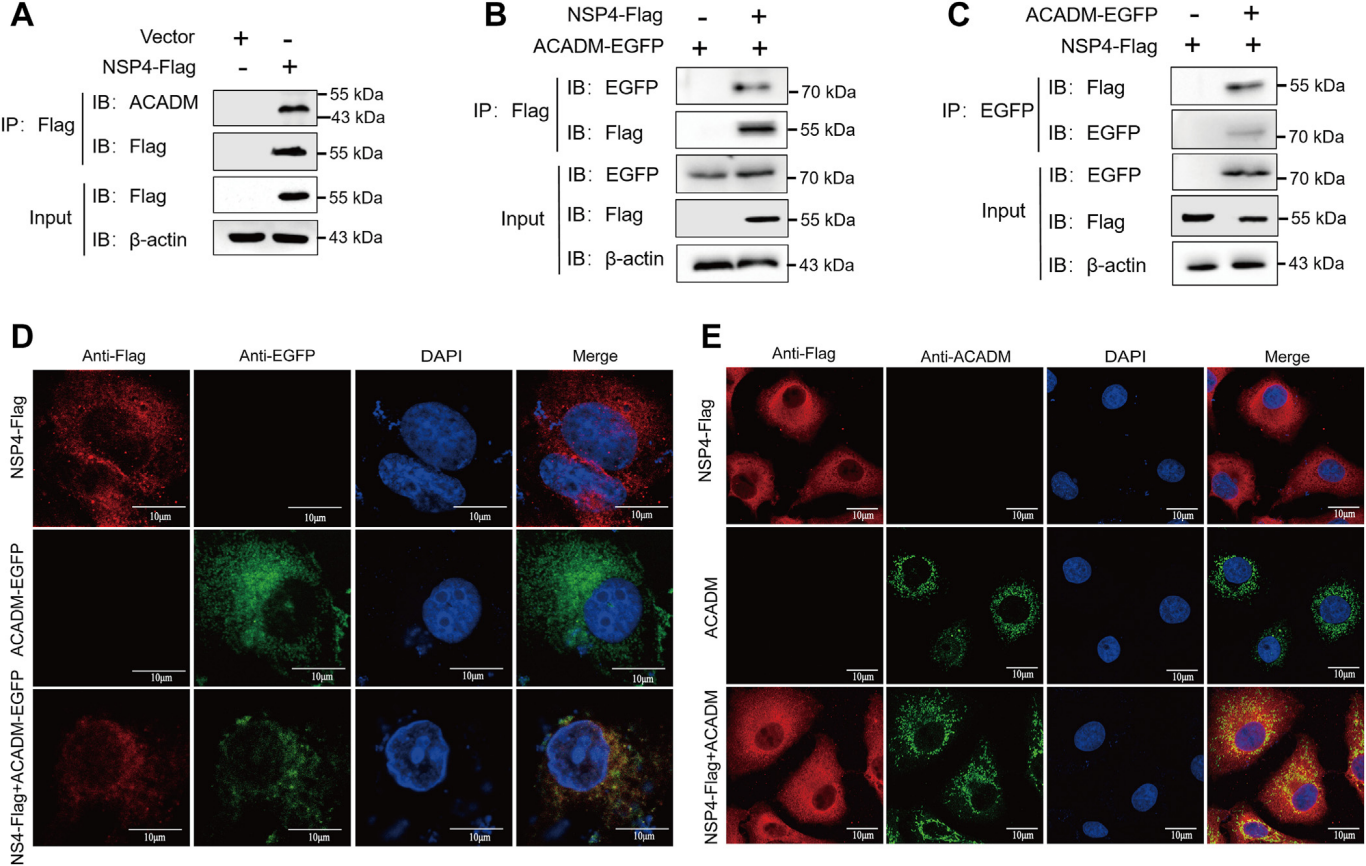
**Interaction between ACADM and PEDV NSP4.***A*, endogenous co-IP validates the binding of ACADM with NSP4. Marc-145 cells were transfected with NSP4-Flag plasmid, and the lysate was analyzed by coimmunoprecipitation (co-IP) and Western blot to detect the interaction between NSP4 and endogenous ACADM. *B* and *C*, exogenous co-IP detects the binding of ACADM with NSP4. Marc-145 cells were transfected with ACADM-EGFP and NSP4-Flag at the same time, the interaction between the two was detected by reciprocal co-IP. *D*, Marc-145 cells were transfected with ACADM-EGFP and NSP4-Flag alone or simultaneously, labeled with specific primary and secondary antibodies and observed fluorescence signal by confocal immunofluorescence microscopy. *E*, Marc-145 cells were transfected with NSP4-Flag plasmid and then labeled with ACADM- and Flag-specific antibodies. The localization of NSP4 and endogenous ACADM was observed by immunofluorescence. Three independent experiments were carried out, the difference between results were calculated by Student’s *t* test, ∗*p* < 0.05; ∗∗*p* < 0.01; and ∗∗∗*p* < 0.001. NSP, nonstructural protein; PEDV, porcine epidemic diarrhea virus.

NSPs play an important role in the pathogenesis of PEDV; the study of PEDV NSP interaction with host to regulate its replication is more and more extensive. To identify the host proteins that interact with PEDV NSP4, we first performed immunoprecipitation-mass spectrometry analysis (Tables S1 and S2). Among these proteins, ACADM attracted our attention and was selected for further study. First, the interaction between NSP4 and endogenous ACADM was confirmed by transfection of NSP4-Flag into Marc-145 cells and immunoprecipitation with anti-Flag (Fig. 1*A*). We then validated the exogenous physical interaction of NSP4 with ACADM by coimmunoprecipitation assay. Marc-145 cells were transfected with NSP4-Flag and ACADM-EGFP, alone or together, using anti-Flag and anti-GFP antibodies for immunoprecipitation. ACADM-EGFP was immunoprecipitated with NSP4 when they were coexpressed but not in the absence of NSP4, indicating that ACADM interacts with NSP4 (Fig. 1*B*), then we did the reverse verification and obtained the same result (Fig. 1*C*). The colocalization of NSP4 and ACADM was further examined using laser confocal microscopy. NSP4-Flag and ACADM-EGFP were transfected into Marc-145 cells to detect protein localization. The results revealed the colocalization of NSP4 and ACADM (Fig. 1*D*), providing additional confirmation of their interaction. At the same time, we also detected the localization of NSP4 and endogenous ACADM, the findings also showed that there was colocalization between NSP4 and ACADM (Fig. 1*E*). Consequently, our results indicate that ACADM serves as a host interaction factor for PEDV NSP4.

### ACADM expression is increased after PEDV infection or NSP4 transfection

**Figure 2:**
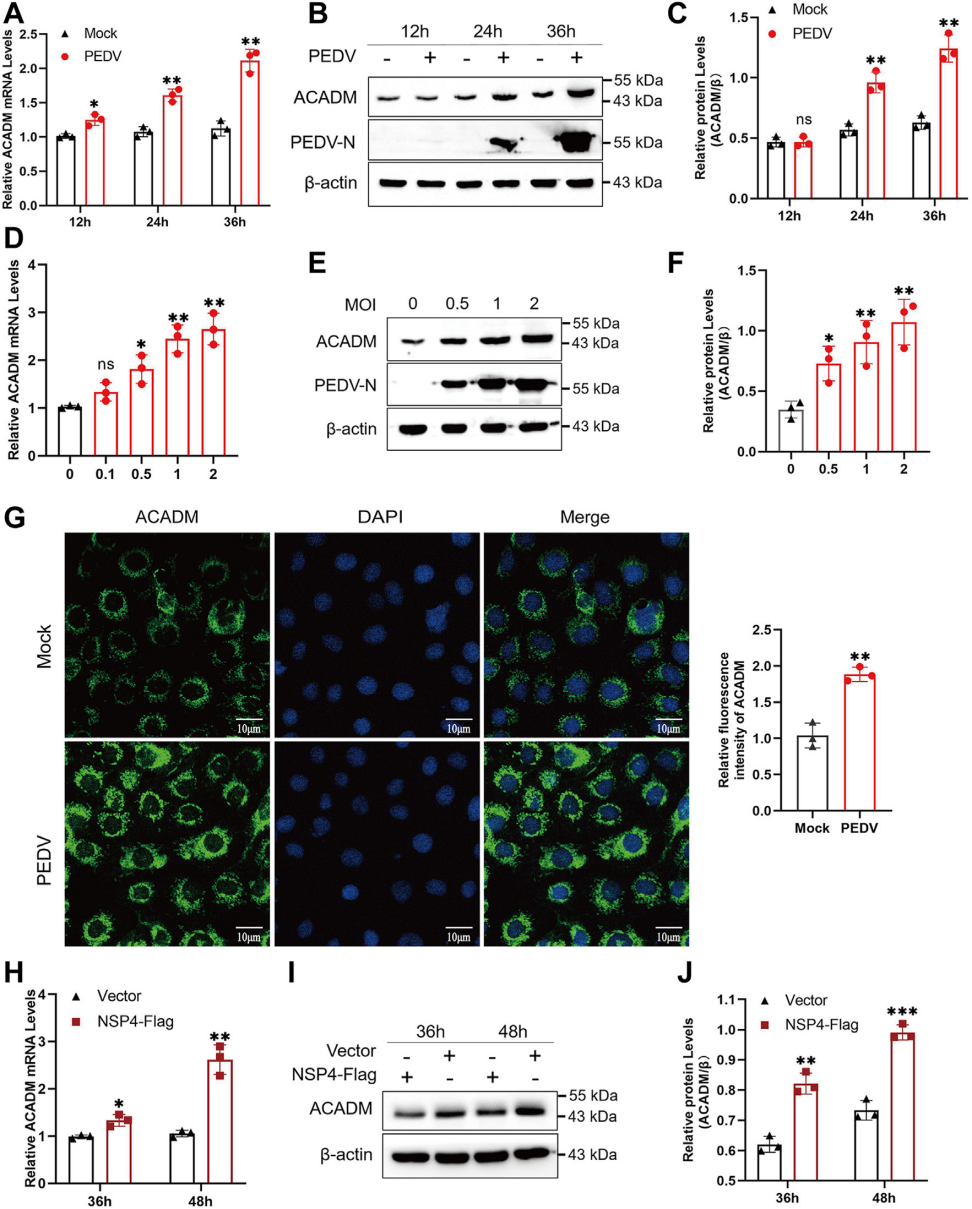
**Changes in expression of ACADM.***A*, Marc-145 cells were infected with PEDV (MOI of 1) or not, the cells were harvested at 12 h, 24 h, and 36 h to detect ACADM mRNA levels by qPCR. *B*, Marc-145 cells were infected with PEDV (MOI of 1) or not, the cells were harvested at 12 h, 24 h, and 36 h to detect ACADM protein levels by Western blot. *C*, the protein levels of ACADM were normalized with β-actin. *D*, Marc-145 cells were infected with PEDV at MOI of 0.1, 0.5, 1, and 2, ACADM mRNA levels were determined after infection 24 h by qPCR. *E*, Marc-145 cells were infected with PEDV at MOI of 0.5, 1, and 2, ACADM protein levels were determined after infection 24 h by Western blot. *F*, the protein levels of ACADM were normalized to β-actin. *G*, Marc-145 cells were infected with PEDV (MOI of 1) or not, the expression of ACADM was detected by immunofluorescence assay. The fluorescence intensity ACADM was analyzed with Image J. *H*, Marc-145 cells were transfected with NSP4-Flag or empty vector for 36 h and 48 h, qPCR was conducted to detect ACADM mRNA levels. *I*, Marc-145 cells were transfected with NSP4-Flag or empty vector for 36 h and 48 h, Western blot was performed to detect ACADM protein levels. *J*, the protein levels of ACADM were normalized with β-actin. All data were obtained from three independently repeated experiments (n = 3), and the difference between results were calculated by Student’s *t* test, ∗*p* < 0.05; ∗∗*p* < 0.01; ∗∗∗*p* < 0.001. MOI, multiplicity of infection; NSP, nonstructural protein; PEDV, porcine epidemic diarrhea virus; qPCR, real-time quantitative PCR.

The interaction between ACADM and NSP4 has been demonstrated, it remains unclear whether PEDV infection or NSP4 transfection affects the expression of ACADM. To address this, we infected Marc-145 cells with PEDV and harvested them at specific time points, the expression of endogenous ACADM were detected by real-time quantitative PCR (qPCR) and Western blot. The results showed that PEDV infection notably increased the mRNA and protein levels of ACADM in a time-dependent manner (Fig. 2, *A*–*C*). We then infected Marc-145 cells with PEDV at different multiplicity of infections (MOIs) for 24 h to determine the expression of ACADM, and it displayed that the mRNA levels and protein levels of ACADM increased in a dose-dependent manner (Fig. 2, *D*–*F*). In order to more intuitively detect the change in ACADM expression, immunofluorescence assays were conducted, revealing an upregulation of ACADM expression following PEDV infection (Fig. 2*G*). Because NSP4 of PEDV interacts with ACADM, we further explored whether NSP4 affects the expression of ACADM. Compared with those in the empty vector–transfected group, the mRNA and protein levels of ACADM in the NSP4-transfected group were significantly increased (Fig. 2, *H*–*J*), which was consistent with the results of PEDV infection. These results indicate that ACADM expression is upregulated following PEDV infection or NSP4 transfection.

### ACADM impairs PEDV replication

**Figure 3:**
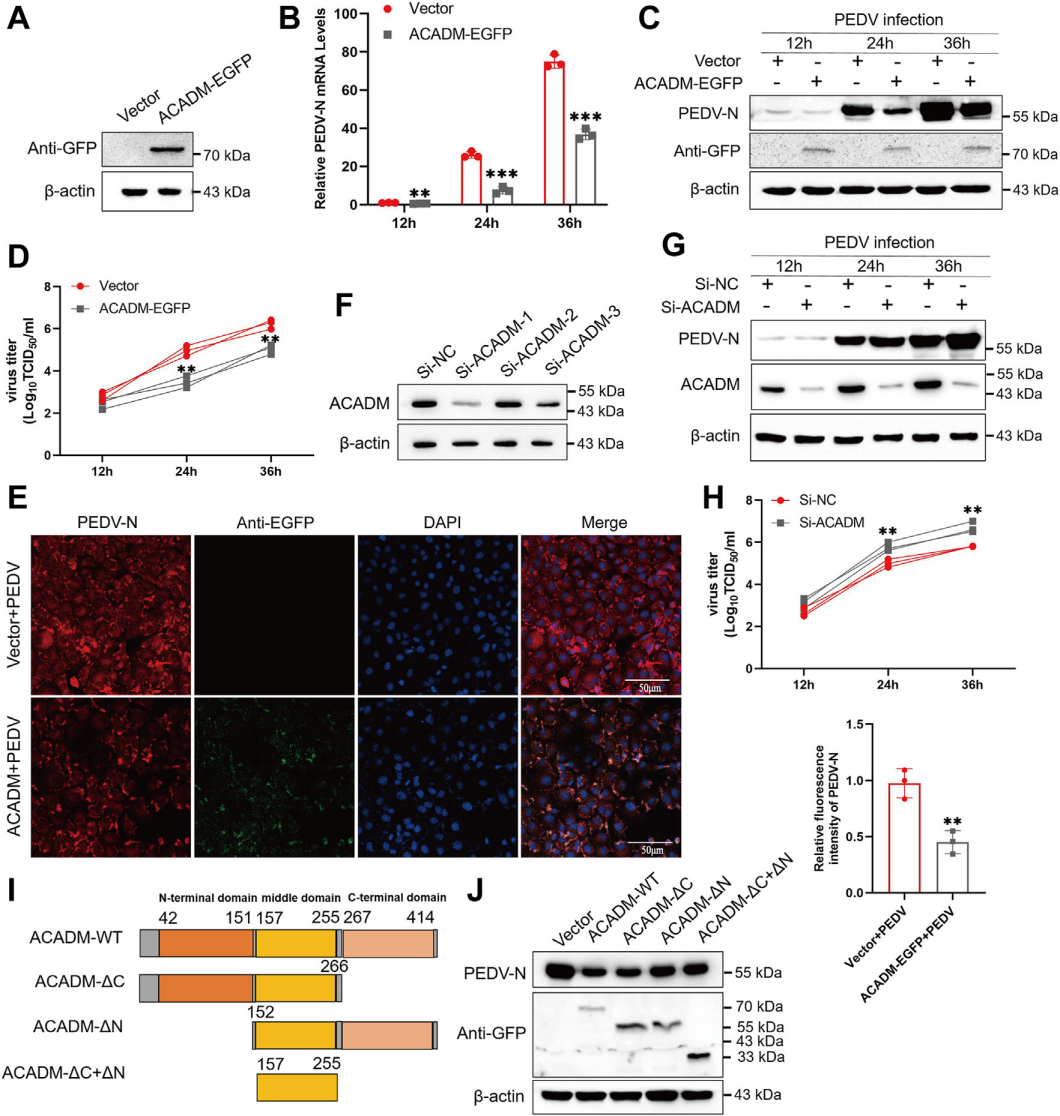
**ACADM affects PEDV replication.***A*, Marc-145 cells were transfected with ACADM-EGFP or empty vector to verify the overexpression efficiency of ACADM-EGFP. *B* and *C*, Marc-145 cells were transfected with ACADM-EGFP or empty vector for 36 h and infected with PEDV at MOI of 1 for 12 h, 24 h, and 36 h. The PEDV N mRNA levels were evaluated by real-time quantitative PCR (*B*) and the PEDV N protein levels were evaluated by Western blot (*C*). *D*, the supernatant was harvested and the virus titer was measured by TCID_50_. *E*, Marc-145 cells were transfected with ACADM-EGFP or empty vector for 36 h and infected with PEDV at 1 MOI for 24 h. The cells were collected to fix and then incubate with mouse anti-PEDV N polyclonal serum (1:500) for overnight at 4 °C. The replication of PEDV in the cells was observed by immunofluorescence assays. The fluorescence intensity of PEDV N (*red*) was analyzed with Image J. *F*, ACADM specific siRNA were transfected into Marc-145 cells for 36 h to assess the knockdown efficiency of ACADM. *G*, Marc-145 cells were transfected with siACADM or siNC for 36 h and infected with PEDV at MOI of 1 for 12 h, 24 h, and 36 h. Western blot was performed to evaluate the PEDV N protein levels. *H*, PEDV titers in supernatant were determined by TCID_50_ method. *I*, the functional region of ACADM, full-length ACADM-EGFP, deletion of C-terminal mutant ACADM-ΔC-EGFP, deletion of N-terminal mutant ACADM-ΔN-EGFP, deletion of both C-terminal, and N-terminal mutant ACADM-ΔC+ΔN-EGFP were constructed. *J*, ACADM-WT-EGFP, ACADM-ΔC-EGFP, ACADM-ΔN-EGFP, and ACADM-ΔC+ΔN-EGFP were solely transfected into Marc-145 cells for 36 h and then PEDV infected at 1 MOI for 24 h. The expression levels of ACADM constructs and PEDV N were detected by Western blot. Each result was obtained from three independent repeated assays, Student’s *t* test was used to calculate the difference, which are denoted as follows: ∗*p* < 0.05; ∗∗*p* < 0.01; and ∗∗∗*p* < 0.001. MOI, multiplicity of infection; PEDV, porcine epidemic diarrhea virus; TCID_50_, median tissue culture infection dose; siNC, small interfering RNA of negative control.

The upregulation of ACADM expression by PEDV infection suggests its potential involvement in PEDV replication. To investigate this, we successfully expressed ACADM-EGFP in Marc-145 cells and confirmed overexpression efficiency by Western blot (Fig. 3*A*). Marc-145 cells were transfected with ACADM-EGFP or empty vector for 36 h and then infected with PEDV. Cells were harvested at different time points to evaluate PEDV N expression and PEDV viral load. qPCR and Western blot results showed that compared with the empty vector, the overexpression of ACADM significantly decreased the mRNA and protein levels of PEDV N (Fig. 3, *B* and *C*), and the results of the median tissue culture infection dose also indicated that overexpression of ACADM decreased the titer of PEDV (Fig. 3*D*). Meanwhile, we conducted indirect immunofluorescence to validate the role of ACADM in PEDV replication. The results confirmed that ACADM overexpression suppressed PEDV replication (Fig. 3*E*). To further ascertain the function of ACADM in PEDV replication, we designed and synthesized specific siRNAs targeting ACADM. These siRNAs were transfected into Marc-145 cells, and their effectiveness was assessed *via* Western blot analysis (Fig. 3*F*). Notably, small interfering RNA of ACADM-1 demonstrated the most prominent inhibitory effect on protein expression, which was selected for subsequent study. Transfection with siACADM significantly promoted PEDV replication compared with the normal control siRNA group (Fig. 3*G*). Likewise, the PEDV titer was elevated in cells transfected with siACADM (Fig. 3*H*). Collectively, our data indicate that ACADM exerts a negative regulatory effect on PEDV replication.

Subsequently, we aimed to further investigate the functional regions of ACADM that hinder PEDV replication by constructing ACADM-ΔC-EGFP, ACADM-ΔN-EGFP, and ACADM-ΔC+ΔN-EGFP (Fig. 3*I*). These constructs were transfected into Marc-145 cells for 36 h, followed by PEDV infection for 24 h. Western blot analysis revealed that the absence of both the N-terminal and C-terminal regions of ACADM still inhibited PEDV replication, suggesting that the critical region of ACADM responsible for inhibiting PEDV replication is located in the middle domain (Fig. 3*J*).

### ACADM inhibits the synthesis of PEDV dsRNA, but has no effect on attachment, entry, and release of PEDV

**Figure 4:**
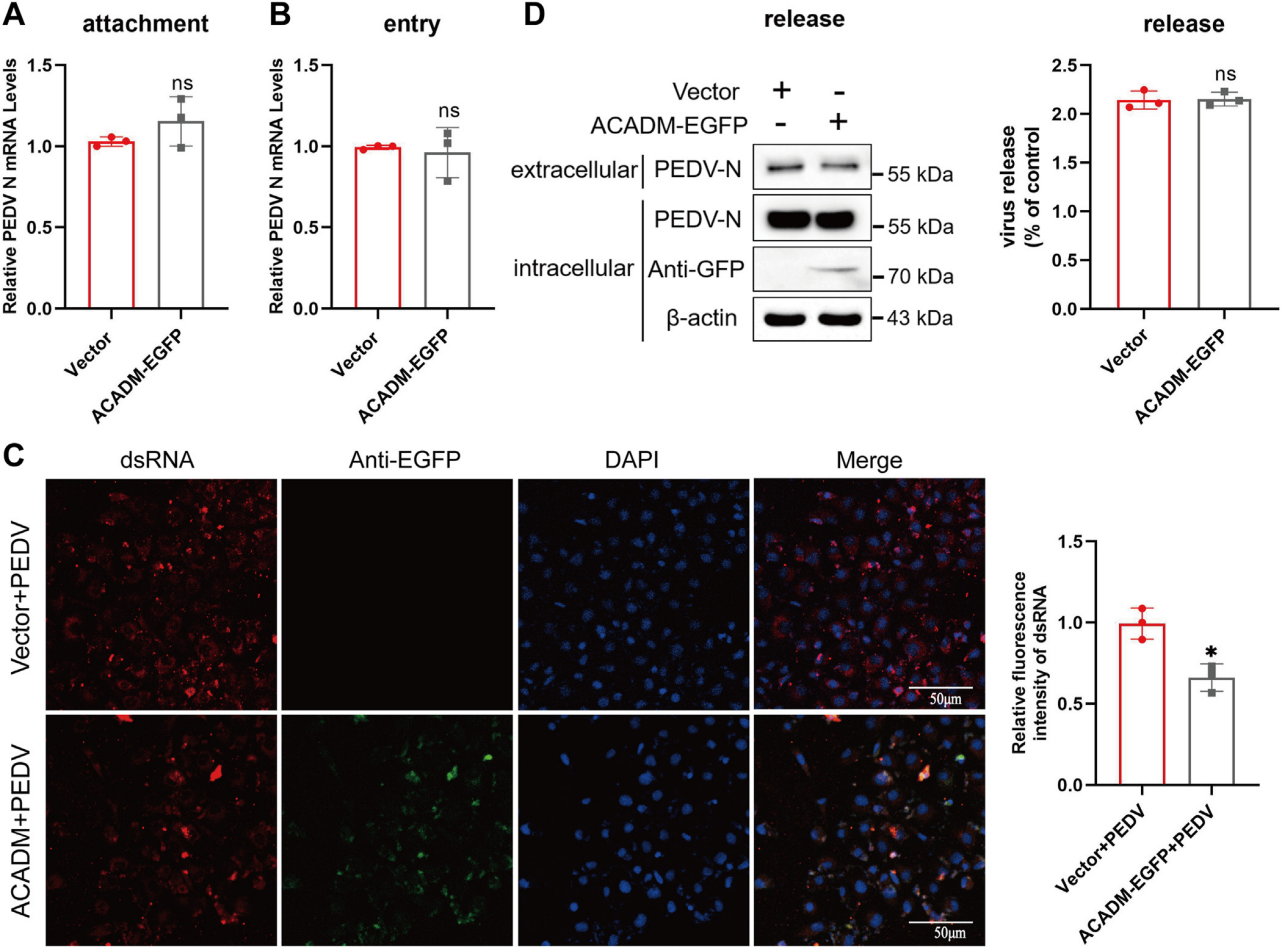
**Overexpression of ACADM inhibits PEDV replication—but has no effect on attachment—entry, or release.***A* and *B*, Marc-145 cells were transiently transfected with ACADM-EGFP or empty vector for 36 h and incubated with PEDV at MOI of 10 for 1.5 h at 4 °C (*A*) or subsequently transferred to 37 °C for 1 h after the unbound virus were removed (*B*) and then detecting the PEDV N mRNA levels. *C*, the content of PEDV dsRNA (*red*) was detected by immunofluorescence assay. Marc-145 cells were overexpressed ACADM and then infected with PEDV at MOI of 10 for 24 h. The cells were fixed and stained with dsRNA and 4′,6-diamidino-2-phenylindole and then observed by confocal microscopy. The fluorescence intensity of dsRNA was analyzed with Image J. *D*, Marc-145 cells were transiently transfected with ACADM-EGFP for 36 h and incubated with PEDV at MOI of 1 for 36 h. Western blot was used to detect PEDV N in cell lysate and supernatant and Image J was used to quantify. The ratio of intracellular and extracellular N levels represented the release efficiency of the virus. The data were obtained from three independently repeated experiments (n = 3) and the difference between results were calculated by Student’s *t* test, ∗*p* < 0.05; ∗∗*p* < 0.01; and ∗∗∗*p* < 0.001. MOI, multiplicity of infection; PEDV, porcine epidemic diarrhea virus.

Having established that ACADM exerts a negative regulatory effect on PEDV replication, we proceeded to investigate the impact of ACADM on each stage of the PEDV life cycle. Marc-145 cells were transfected with ACADM-EGFP or empty vector for 36 h and then incubated with PEDV at MOI of 10 for 1.5 h at 4 °C. Viral attachment was evaluated using qPCR, revealing no significant differences in viral attachment between ACADM-EGFP–expressing cells and empty vector–expressing cells (Fig. 4*A*). To determine whether overexpression of ACADM affects viral entry, Marc-145 cells were shifted from 4 °C to 37 °C for 1.5 h after removal of unbound virions, the results showed no significant difference between the overexpression group and the control group (Fig. 4*B*). To assess PEDV replication, Marc-145 cells were transfected with either ACADM-EGFP or empty vector and then infected with PEDV for 24 h. Following this, the cells were fixed and stained for dsRNA, a marker for viral replication. Our examination revealed a significant inhibition of dsRNA synthesis upon ACADM overexpression (Fig. 4*C*). Subsequently, virus release was evaluated by Western blot analysis of intracellular and extracellular N protein levels in cells overexpressing ACADM and those with the empty vector, showing no significant disparity (Fig. 4*D*). Therefore, we conclude that ACADM overexpression impedes viral replication by affecting dsRNA synthesis during the PEDV life cycle, while it does not affect viral attachment, entry, or release.

### Fatty acid β-oxidation is essential for PEDV replication

**Figure 5:**
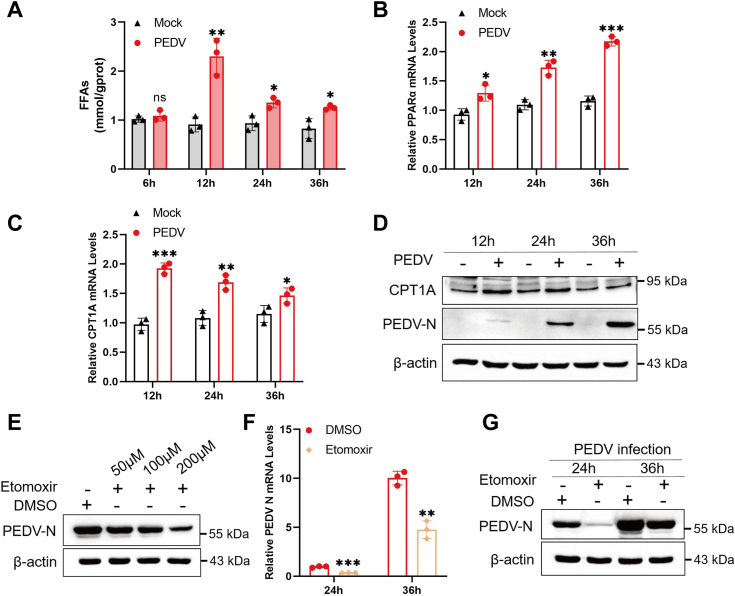
**Fatty acid β-oxidation is crucial for PEDV replication.***A*, Marc-145 cells were mock-infected or infected with PEDV at an MOI of 1 for 6 h, 12 h, 24 h, and 36 h, free fatty acids (FFAs) levels were quantified by FFA detection kit. *B* and *C*, Marc-145 cells were mock-infected or infected with PEDV at an MOI of 1 for 12 h, 24 h, and 36 h, the levels of PPARα and CPT1A were determined by real-time quantitative PCR. *D*, PEDV infected Marc-145 cells for 12 h, 24 h, and 36 h, CPT1A protein levels were determined by Western blot. *E*, impact of different doses of Etomoxir treatment on PEDV replication. Marc-145 cells were treated with 50 μm, 100 μm, 200 μm Etomoxir, and infected with PEDV at MOI of 1 for 24 h. Western blot to determine the levels of PEDV N protein. *F* and *G*, impact of Etomoxir treatment on PEDV replication at different time points. Marc-145 cells were treated with 200 μm Etomoxir and infected with PEDV at MOI of 1 for 24 h and 36 h. The cells were harvested to detect PEDV N mRNA (*F*) and protein levels (*G*). All data shown represent results from at least three independent experiments and the difference between results were calculated by Student’s *t* test, ∗*p* < 0.05; ∗∗*p* < 0.01; and ∗∗∗*p* < 0.001. CPT1A, carnitine palmitoyltransferase 1A; MOI, multiplicity of infection; PEDV, porcine epidemic diarrhea virus; PPARα, peroxisome proliferation–activated receptor-α.

Given that ACADM is an enzyme involved in fatty acid β-oxidation, numerous studies have demonstrated that fatty acid β-oxidation serves as an energy source for viral replication (18, 19). Therefore, we wanted to determine whether β-oxidation also plays a role in PEDV replication, but there have not been any reports on this topic. FFAs are transported into mitochondria for β-oxidation. Initially, we examined the levels of intracellular FFAs at various time points during PEDV infection. The findings revealed a significant increase in intracellular FFAs levels following PEDV infection compared to the mock group (Fig. 5*A*). Subsequently, we assessed the expression of peroxisome proliferation–activated receptor-α and carnitine palmitoyltransferase 1A (CPT1A), key genes involved in fatty acid β-oxidation, *via* qPCR. The results demonstrated a significant upregulation in the mRNA levels of both peroxisome proliferation–activated receptor-α and CPT1A after PEDV infection (Fig. 5, *B* and *C*). Further, the protein levels of CPT1A were detected by Western blot and the results were consistent with the mRNA (Fig. 5*D*).

To further investigate the role of β-oxidation in PEDV replication, we treated Marc-145 cells with Etomoxir, a well-known inhibitor of fatty acid β-oxidation. 3-(4,5-dimethyl-2-thiazolyl)-2,5-diphenyl-2-H-tetrazolium bromide (MTT) assays showed that Etomoxir treatment did not cause any significant cytotoxicity to cells (Fig. S1). Cells were treated with Etomoxir of 50 μm,100 μm, and 200 μm, respectively, and then infected with PEDV at MOI of 1 for 24 h. Western blot analysis was performed to determine PEDV N protein expression, and it was observed that different doses of Etomoxir treatment significantly reduced PEDV N protein levels (Fig. 5*E*). Then, after treatment with 100 μM Etomoxir, the cells were collected at 24 h and 36 h after PEDV infection. qPCR and Western blot results revealed that Etomoxir treatment also impaired PEDV replication at different time points (Fig. 5, *F* and *G*). In summary, these findings suggest that PEDV infection induces fatty acid β-oxidation. Inhibiting β-oxidation effectively suppresses PEDV replication, highlighting the significance of this process in PEDV proliferation and its role as a vital energy source for efficient viral replication.

### ACADM inhibits PEDV-induced fatty acid β-oxidation

**Figure 6:**
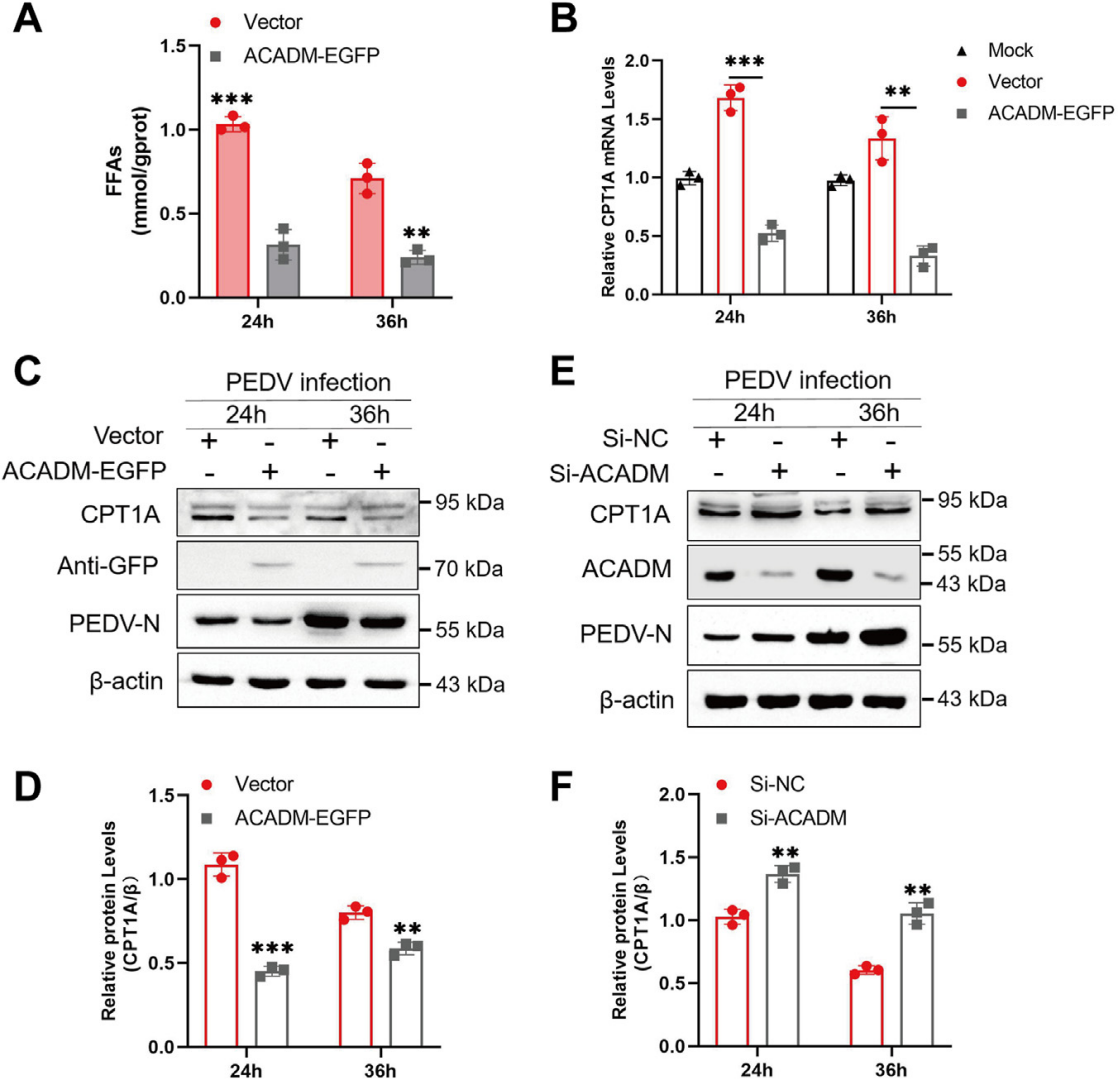
**ACADM negatively regulates PEDV-induced β-oxidation.***A*, Marc-145 cells of overexpressing ACADM were infected with PEDV at MOI of 1 for 24 h and 36 h, using free fatty acid detection kit to detect the levels of FFAs in the cells. *B*, Marc-145 cells overexpressing ACADM were infected with PEDV at MOI of 1 for 24 h and 36 h, the CPT1A mRNA levels were detected by real-time quantitative PCR. *C*, Marc-145 cells were overexpressed ACADM and then infected with PEDV at MOI of 1 for 24 h and 36 h, the CPT1A protein levels were detected by Western blot. *D*, CPT1A protein levels were normalized using β-actin. *E*, Marc-145 cells were infected with PEDV at MOI of 1 for 24 h and 36 h after knocking down ACADM, the CPT1A protein levels were determined by Western blot. *F*, CPT1A protein levels were normalized using β-actin. Each result was obtained from three independent repeated assays, Student’s *t* test was used to calculate the difference, which are denoted as follows: ∗, *p* < 0.05; ∗∗, *p* < 0.01; and ∗∗∗, *p* < 0.001. CPT1A, carnitine palmitoyltransferase 1A; MOI, multiplicity of infection; PEDV, porcine epidemic diarrhea virus.

ACADM has been documented to possess tumor-inhibiting capabilities in hepatocellular carcinoma (HCC). It inhibits fatty acid oxidation mediated by ACADM, promoting HCC through CAV1/SREBP1 signaling (20). To ascertain whether ACADM influences PEDV-induced fatty acid β-oxidation and consequently inhibits virus replication, we assessed FFAs levels in ACADM-transfected cells. We discovered that compared with empty vector treatment, the overexpression of ACADM significantly decreased PEDV-induced FFA levels (Fig. 6*A*). Simultaneously, we conducted qPCR and Western blot to analyses the expression of CPT1A. It was observed that the overexpression of ACADM significantly inhibited the PEDV-induced increase in CPT1A expression (Fig. 6, *B*–*D*). Conversely, transfection with siACADM, aimed at knocking down ACADM expression, markedly enhanced PEDV-induced CPT1A expression (Fig. 6, *E* and *F*). These findings conclusively demonstrate that ACADM impedes PEDV-induced fatty acid β-oxidation.

### ACADM negatively regulates PEDV-induced lipophagy

**Figure 7:**
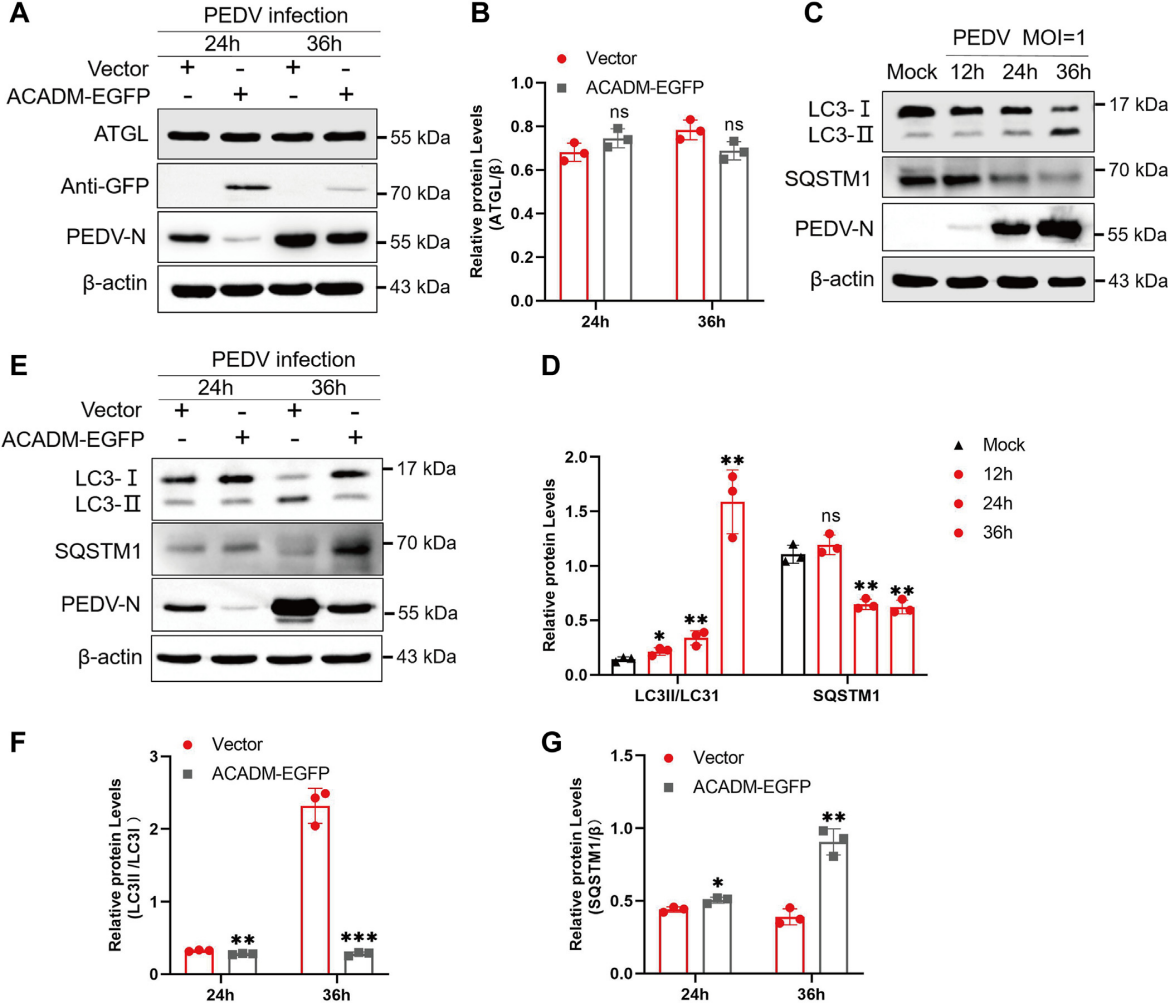
**ACADM impairs PEDV-induced lipophagy.***A*, Marc-145 cells were overexpressed ACADM and then infected with PEDV at MOI of 1 for 24 h and 36 h to examine ATGL protein levels. *B*, the protein levels of ATGL was normalized to β-actin. *C*,Western blot analysis the autophagy activation in PEDV-infected cells, Marc-145 cells were infected with PEDV at MOI of 1 for 12 h, 24 h, and 36 h to detect the expression levels of LC3 and SQSTM1. *D*, the protein levels of LC3II were normalized to LC3I, and the protein levels of SQSTM1 were normalized to β-actin. *E*, Western blot analysis of LC3 and SQSTM1 expression in Marc-145 cells and ACADM-EGFP–expressing cells. *F* and *G*, the protein levels were normalized. All results from at least three independent experiments and the difference between results were calculated by Student’s *t* test, ∗*p* < 0.05; ∗∗*p* < 0.01; and ∗∗∗*p* < 0.001. ATGL, adipose triglyceride lipase; LC3, light chain 3; MOI, multiplicity of infection; PEDV, porcine epidemic diarrhea virus; SQSTM1, sequestosome-1.

Triglycerides are released from LDs and hydrolyzed to FFAs through two different mechanisms, lipolysis and lipophagy, and then drive mitochondrial β-oxidation (21, 22). Having shown that the overexpression of ACADM inhibits the PEDV-induced increase in FFAs and β-oxidation, our next aim is to determine which of these two mechanisms regulates this process. Marc-145 cells were transfected with ACADM-EGFP or empty vector and then subjected to PEDV infection. Western blot was used to determine the expression level of the lipolysis rate-limiting enzyme adipose triglyceride lipase, no significant difference was found between the two groups (Fig. 7, *A* and *B*). Concurrently, we assessed the expression levels of autophagy activation markers, including microtubule-associated protein light chain 3 (LC3) and sequestosome-1. The results indicated that PEDV infection activated autophagy (Fig. 7, *C* and *D*). However, the overexpression of ACADM inhibited PEDV-induced autophagy (Fig. 7, *E*–*G*).

**Figure 8:**
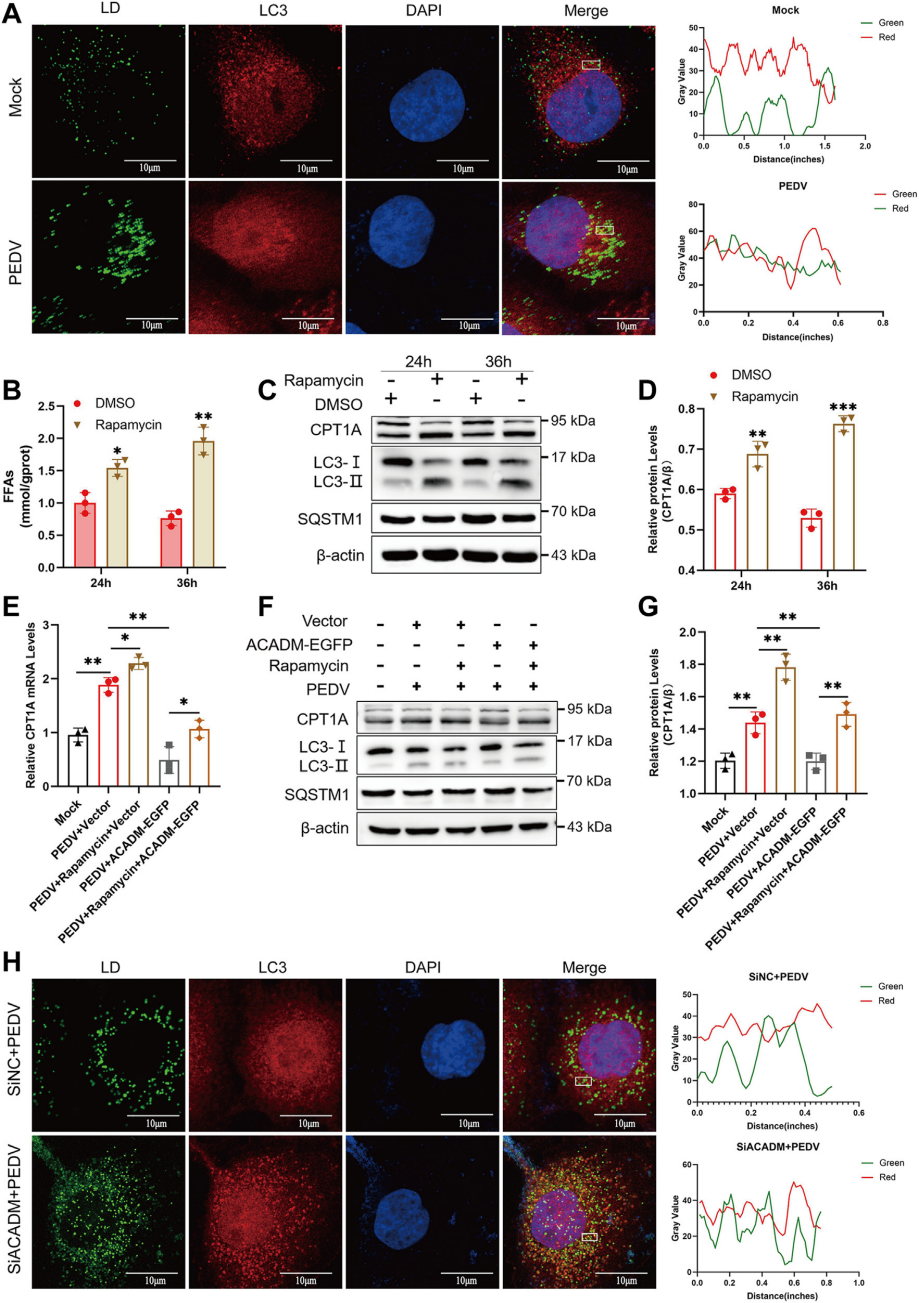
**ACADM impairs PEDV-induced lipophagy.***A*, Marc-145 cells were mock-infected or infected with PEDV at MOI of 1 for 36 h, the cells were harvested for fixation and staining. Confocal microscopy was used to observe the location of LDs and LC3 in mock group and PEDV-infected group. *B*, the cells were pretreated with the autophagy activator rapamycin for 1 h and then infected with PEDV at MOI of 1 for 24 h and 36 h, the intracellular FFAs levels were examined. *C*, the cells were infected with PEDV at 1 MOI for 24 h and 36 h after pretreatment with rapamycin, the expression levels of CPT1A were measured by Western blot. *D*, the intensity represents CPT1A protein levels normalized to the β-actin. *E* and *F*, Marc-145 cells overexpressed with ACADM were pretreated with rapamycin and then infected with PEDV at MOI of 1 for 36 h. The mRNA and protein levels of CPT1A were detected by real-time quantitative PCR (*E*) and Western blot (*F*), respectively. *G*, the CPT1A protein levels were normalized to β-actin. *H*, Marc-145 cells were transfected with siACADM or siNC for 36 h and then infected PEDV for 36 h. The cells were collected for fixing and specific staining to examine the colocalization of LC3 and LDs. The results represent three independent trials, the difference was measured by Student’s *t* test, ∗*p* < 0.05; ∗∗*p* < 0.01; and ∗∗∗*p* < 0.001. CPT1A, carnitine palmitoyltransferase 1A; FFA, free fatty acid; LC3, light chain 3; LD, lipid droplet; MOI, multiplicity of infection; PEDV, porcine epidemic diarrhea virus; siNC, small interfering RNA of negative control.

To gain further insight into the induction of lipophagy during PEDV infection, we employed laser confocal microscopy to analyze the colocalization between LC3 and LD in PEDV-infected cells. We found that PEDV infection enhanced the fluorescence signals of LC3 and LDs compared with those in mock cells and colocalization of LC3 and LDs occurred (Fig. 8*A*). Moreover, in order to better confirm the relationship between autophagy and LD degradation during PEDV infection, we treated cells with the autophagy activator rapamycin. After PEDV infection, the levels of FFAs and the expression of the β-oxidation rate-limiting enzyme CPT1A in cells were detected. Compared with those in the dimethyl sulfoxide treatment group, the activation of autophagy significantly increased the level of intracellular FFAs and the expression of CPT1A (Fig. 8, *B*–*D*).

To gain a deeper understanding of how ACADM suppresses PEDV-induced FFAs and β-oxidation by inhibiting lipophagy, we conducted a rescue experiment using rapamycin. Cells overexpressing ACADM-EGFP were treated with Rapamycin. qPCR and Western blot results revealed that rapamycin treatment restored the inhibitory effect of ACADM on CPT1A expression, but this phenomenon was not observed in the dimethyl sulfoxide treatment group (Fig. 8, *E*–*G*). Moreover, Marc-145 cells were transfected with siACADM and then infected with PEDV for 36 h. After fixation and staining, the cells were examined using laser confocal microscopy. The findings demonstrated a significant increase in the colocalization of LC3 and LDs upon ACADM knockdown (Fig. 8*H*). In conclusion, these evidences indicate that PEDV infection can induce lipophagy, but ACADM negatively regulates PEDV-induced lipophagy, thereby inhibiting intracellular FFAs levels and fatty acid β-oxidation.

### ACADM suppresses AMPK activation to inhibit PEDV-induced lipophagy

**Figure 9:**
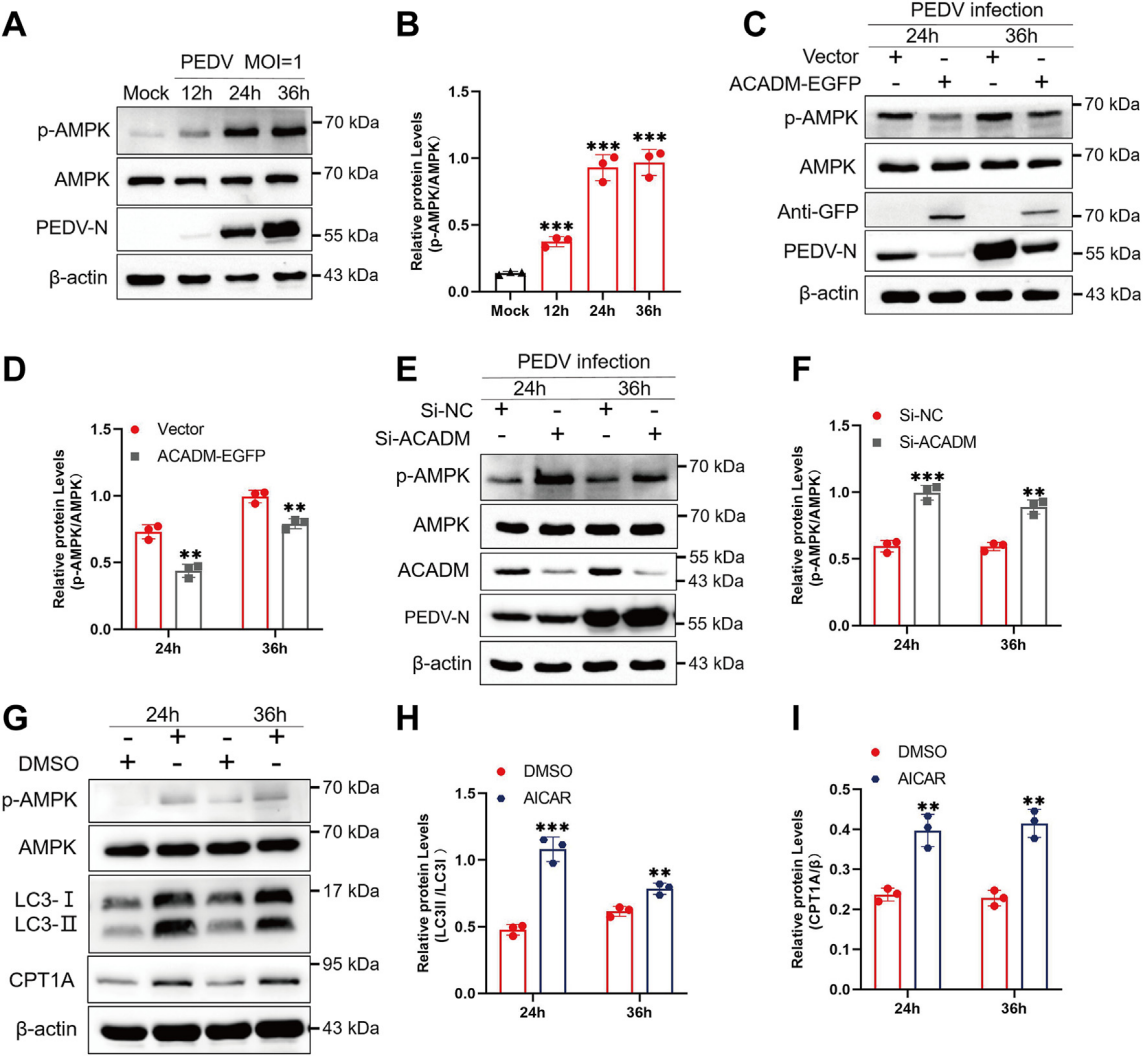
**ACADM inhibits PEDV-induced AMPK activation to inhibit lipophagy.***A*, PEDV infection activates AMPK. Marc-145 cells were infected with PEDV for 12 h, 24 h, and 36 h, Western blot analysis of p-AMPK, and AMPK protein levels. *B*, the intensity shows that p-AMPK were normalized to AMPK. *C*, the effect of ACADM overexpression on AMPK activation. Western blot analysis of the expression levels of p-AMPK and AMPK in empty vector–expressing cells and ACADM-EGFP–expressing cells. *D*, the intensity represent that p-AMPK was normalized to AMPK. *E* and *F*, after knocking down the expression of ACADM in Marc-145 cells and then infected with PEDV at 1 MOI. The changes of p-AMPK and AMPK were detected by Western blot and p-AMPK was normalized to AMPK. *G*, the cells were pretreated with AMPK activator ACIAR for 1 h and infected with PEDV at MOI of 1. The expressions of LC3 and CPT1A were measured by Western blot. *H* and *I*, the protein levels were normalized. The data were obtained from three independent assays, Student’s *t* test was used to calculate the difference, which are denoted as follows: ∗*p* < 0.05; ∗∗*p* < 0.01; and ∗∗∗*p* < 0.001. CPT1A, carnitine palmitoyltransferase 1A; LC3, light chain 3; MOI, multiplicity of infection; PEDV, porcine epidemic diarrhea virus.

AMPK is an intracellular energy sensor that can regulate autophagy and lipid metabolism (23), this led us to explore whether AMPK plays a role in the process by which ACADM inhibits lipophagy. Initially, we examined the alterations in AMPK following PEDV infection *via* Western blot analysis. The results revealed a significant increase in p-AMPK expression post-PEDV infection, indicative of AMPK activation (Fig. 9, *A* and *B*). Then Marc-145 cells were transfected with ACADM-EGFP for 36 h followed by PEDV infection. AMPK activation was detected by Western blot, which found that overexpression of ACADM significantly suppressed PEDV-induced AMPK activation (Fig. 9, *C* and *D*). Conversely, knocking down ACADM resulted in a contrasting outcome, transfection with siACADM notably augmented AMPK activation compared to small interfering RNA of negative control (Fig. 9, *E* and *F*). To further substantiate that ACADM suppresses PEDV-induced lipophagy by inhibiting AMPK activation, thereby reducing intracellular FFAs and subsequent β-oxidation, cells were pretreated with the AMPK activator 5-aminoimidazole-4-carboxamide1-β-D-ribofuranoside (AICAR) before PEDV infection, and the protein levels of LC3 and CPT1A were assessed. The results demonstrated that AICAR treatment significantly increased the expression of LC3II/LC3I and CPT1A (Fig. 9, *G*–*I*). In summary, these findings underscore that ACADM suppresses AMPK activation, thereby negatively regulating β-oxidation to impede PEDV replication.

## Discussion

Positive ssRNA viruses have the ability to remodel the host cell membrane, inducing the formation of double-membrane vesicles. This process facilitates RNA replication and helps the virus evade the host immune response (24). Previous studies have shown that coronavirus NSP4 plays a role in double-membrane vesicle formation, underscoring its importance in virus replication (25, 26, 27). Despite this, research on PEDV NSP4 is limited, making it crucial to analyze host proteins interacting with NSP4 to better understand PEDV pathogenesis. In this study, we constructed an NSP4-Flag eukaryotic expression plasmid and introduced it into Marc-145 cells. Using immunoprecipitation-mass spectrometry, we further analyzed the host NSP4- interacting protein and demonstrated that ACADM is a host NSP4-interacting partner *via* coimmunoprecipitation and confocal immunofluorescence.

As a lipid metabolism-related enzyme, ACADM is involved in fatty acid β-oxidation. Abnormal ACADM can block the metabolism of medium-chain fatty acids, reduce the production of acetyl-CoA, and decrease the production of ATP (28). ACADM has been linked to metabolic diseases, cancer, and pathogen infections and changes in its expression were also observed in theses process. For example, ACADM was significantly expressed at low levels in HCC, and suppression of ACADM could increase the levels of triglycerides, phospholipids, and LDs, indicating that it has a tumor inhibitory effect on HCC (20). ACADM was reduced in mouse livers infected with *chlamydia* pneumoniae, thus affecting cholesterol and triglyceride metabolism (29). In mammary epithelial cells, miR-224 has been found to downregulate ACADM expression, affecting apoptosis and triglyceride production (30). Nonetheless, the function of ACADM in PEDV infection remains unexplored. Our study marks the first report of the interaction between PEDV NSP4 and ACADM. We observed that both PEDV infection and NSP4 transfection led to an increase in intracellular ACADM expression, with effects dependent on time and dosage. These findings suggest that PEDV may influence ACADM expression through NSP4. Furthermore, our experiments involving ACADM overexpression and knockdown revealed its negative regulatory role in PEDV replication.

Viruses rely on cellular mechanisms to proliferate, causing alterations in host cellular metabolism, including glycolysis, amino acid metabolism, lipid synthesis, and lipid catabolism, creating an optimal environment for replication (31, 32, 33, 34). Intracellular lipids are vital in viral infection processes, various viruses like HCV (35), DENV (36), severe acute respiratory syndrome coronavirus 2 (37), classical swine fever virus (38), rabies virus (39), among others, boost their proliferation by upregulating lipid synthesis. Moreover, viruses such as DENV (11), enteroviruses (18, 40), *etc*. can mobilize FFAs from LDs, enhancing mitochondrial fatty acid oxidation to fuel viral replication. However, whether and how PEDV utilizes fatty acid β-oxidation to provide energy for its replication has not been reported. Our study revealed that PEDV infection significantly elevates intracellular FFAs levels alongside mRNA and protein levels of the β-oxidation rate-limiting enzyme CPT1A. Furthermore, the cells were treated with Etomoxir, which is a fatty acid β-oxidation inhibitor. The inhibition of β-oxidation significantly impaired PEDV replication. Our results show for the first time that fatty acid β-oxidation provides energy for PEDV replication, which is consistent with other reports. Interestingly, HCV inhibits mitochondrial β-oxidation to alter cellular energy metabolism and the antiviral response during infection, which affects the health of the host in favor of its pathopoiesis (12). Moreover, for Marek’s disease virus, fatty acid β-oxidation is not necessary for its effective replication (32). These studies suggest that the relationships between different viruses and fatty acid β-oxidation in host cells vary, which may be caused by the different pathogenic mechanisms of viruses in the host.

Autophagy, a process reliant on the lysosomal pathway, serves to degrade cellular damaged organelles, misfolded proteins, and pathogens, essential for maintaining intracellular energy balance and cellular homeostasis (41). Recent investigations have underscored autophagy as a critical mechanism for intracellular LDs degradation. This process, known as lipophagy, entails the degradation of LDs, releasing FFAs into mitochondria for β-oxidation, a vital step in ATP production (42). In this research, we uncovered a novel role of ACADM in modulating host autophagy and lipid metabolism during PEDV infection. Several studies have elucidated the utilization of lipophagy by viruses to generate ATP, meeting their energy demands during the infection cycle. Porcine reproductive and respiratory syndrome virus infection downregulates NDRG1 expression to promote autophagy and increase FFAs levels, which facilitates viral replication (43). White spot syndrome virus infection induces Mindin expression in shrimp. Mindin activates autophagy to induce LDs consumption and triglycerides are hydrolyzed to FFAs, which ultimately provide energy for white spot syndrome virus infection (44). Previous findings from our laboratory have shown that PEDV induces autophagy through the PERK and IRE1 pathways to facilitate its replication, indicating that autophagy plays an active role in PEDV replication (45). Similarly, we observed that overexpression of ACADM inhibited PEDV-induced autophagy, leading to a decrease in the intracellular FFAs content and β-oxidation. The use of autophagy activators can compensate for the reduction in β-oxidation caused by ACADM, suggesting that there is an interrelationship between autophagy and lipid consumption. Moreover, knockdown of ACADM increased the colocalization of LDs with LC3 and enhanced β-oxidation. These findings indicate that ACADM can suppress viral replication by inhibiting autophagy to negatively regulate PEDV-induced increase in FFAs and fatty acid β-oxidation. Our study is consistent with previous studies. Interestingly, ACADM is an enzyme involved in fatty acid β-oxidation reactions. However, the findings of this study revealed that the overexpression of ACADM inhibited PEDV-induced β-oxidation. We suggest that this may be a host strategy to restrict viral replication. Both the virus and the host are in competition with one another. The virus uses the host's materials and energy to infect it, while the host continuously activates its defensive system to fend off virus proliferation. There is a general feedback regulation in these processes (46). And the effect of ACADM on β-oxidation is indirect. Moreover, β-oxidation is a complex enzymatic reaction involving multiple enzymes. Despite consulting numerous articles on ACADM research, there is a scarcity of studies focusing on the specific enzymatic reactions catalyzed by ACADM. Further research is needed to determine if the enzyme activity of ACADM is related to the inhibition of PEDV.

AMPK functions as an essential intracellular regulator of metabolism and energy, influencing various physiological processes such as cell growth, catabolism, anabolism, autophagy, and mitochondrial homeostasis (47). Given its crucial role, many viruses exploit AMPK to manipulate processes like autophagy, lipid metabolism, and glucose metabolism to facilitate their replication (23). For example, in DENV infection, AMPK activation is necessary for inducing lipophagy, supporting viral replication (48). Similarly, Zika virus triggers lipophagy and regulates this process through the AMPK–ULK1–Ser556 signaling pathway (49). PEDV infection induces autophagy and enhances viral replication by activating AMPK and c-Jun N-terminal kinase (50). In our study, we further establish the association between PEDV-induced lipophagy and AMPK activation, demonstrating that ACADM inhibits lipophagy by suppressing AMPK activation.

**Figure 10:**
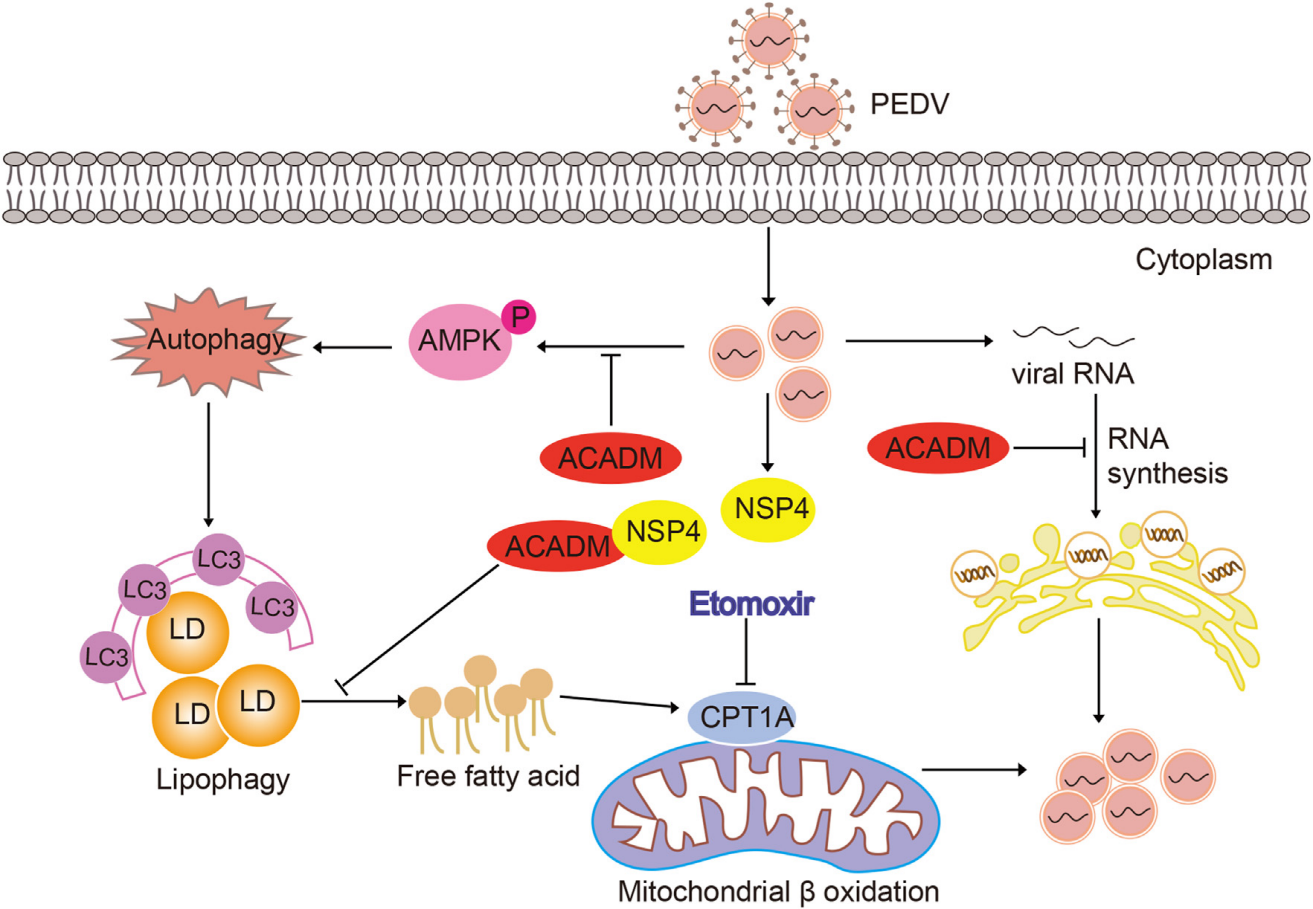
**The mechanism model of ACADM impairs PEDV replication in this study.** ACADM is a host interaction factor of PEDV NSP4. Following PEDV infection or NSP4 expression, ACADM expression is upregulated. Overexpression of ACADM inhibits PEDV dsRNA synthesis. The entry of FFAs into mitochondria for β-oxidation is crucial for PEDV replication. However, ACADM suppresses PEDV-induced AMPK activation and lipophagy, resulting in reduction of FFAs and subsequent β-oxidation, which ultimately impairs PEDV replication. FFA, free fatty acid; NSP, nonstructural protein; PEDV, porcine epidemic diarrhea virus.

In summary, we have outlined a model illustrating the role of ACADM during PEDV infection (Fig. 10). ACADM was identified as a host-interacting protein of PEDV NSP4, and both PEDV infection and NSP4 transfection led to increase the expression of ACADM expression. Furthermore, we observed that PEDV infection promoted β-oxidation by activating AMPK, inducing lipophagy. Inhibition of β-oxidation impaired PEDV replication, suggesting that PEDV may use host β-oxidation to provide energy for its replication. Mechanistically, ACADM suppressed AMPK activation, thereby inhibiting PEDV-induced lipophagy, reducing intracellular FFAs levels and β-oxidation, ultimately negatively regulating PEDV replication. Our findings elucidate a novel mechanism by which ACADM regulates PEDV replication and affirm ACADM as an antiviral factor against PEDV. This sheds light on how PEDV exploits host lipid metabolism for its proliferation and offers new insights for the prevention and control of PEDV infection.

## Experimental procedures

### Cell culture

Monkey embryonic kidney epithelial cells (Marc-145 cells, stored in our laboratory) were cultured in Dulbecco minimal essential medium (HyClone) supplemented with 10% fetal bovine serum (Gibco). Marc-145 cells were grown in incubated at 37 °C with 5% CO_2_.

### Virus, virus titration, and virus infections

The PEDV strain CH/SXYL/2016 was isolated and stored in our laboratory ((51). Ninety six–well plates were covered with monolayer Marc-145 cells then infected with continuously diluted PEDV (10^−1^–10^−10^). Then use the Reed–Muench method calculate the virus titers and transformed into the median tissue culture infection dose. Marc-145 cells were infected PEDV with a MOI of 1.

### Plasmid construction

The pEGFP-N1 empty vector was stored in Laboratory of Veterinary Microbiology, College of Veterinary Medicine, Northwest A&F University. The ACADM-EGFP, ACADM-ΔC-EGFP, ACADM-ΔN-EGFP, and ACADM-ΔC+ΔN-EGFP eukaryotic expression plasmids were constructed. The sequences of ACADM, ACADM-ΔC, ACADM-ΔN, and ACADM-ΔC+ΔN genes were amplified using complementary DNA as a template. The amplification products were digested with restriction enzyme (New England Biolabs) and cloned into the expression vectors using T4 DNA Ligase (EL0011, Thermo Fisher Scientific). All constructed plasmids as described above were confirmed by sequencing. The primers are provided in Table S3.

### Reagents and antibodies

The β-oxidation inhibitor Etomoxir (HY-50202), autophagy activator rapamycin (HY-10219), and AMPK activator AICAR (HY-13417) were obtained from MCE. The polyclonal antibody of against PEDV N was stored in our laboratory (52). Antibodies against β-actin (AC026), CPT1A (A5307), and sequestosome-1 (A19700) were obtained from ABclonal. Antibodies against GFP (AB0005), ACADM (CY5285), LC3 (CY5992), AMPK (CY5326), and p-AMPK (CY5608) were obtained from Abways. Antibody against Flag (HT201-01) was obtained from TransGen Biotech. Mouse mAb against dsRNA J2 was obtained from Nordic MUbio (SCICONS). Peroxidase AffiniPure Goat Anti-Rabbit IgG (DY60202) and anti-mouse IgG (DY60203) were obtained from Diyi Biotechnology. The siRNA sequence was designed and synthesized from General Biol Company.

### RNA extraction and quantitative real-time PCR

Total RNA was extracted from Marc-145 using TRIzol Universa (TIANGEN). It was then reverse-transcribed into complementary DNA using the FastKing RT Kit (TIANGEN) according to the manufacturer’s instructions; qPCR analysis was performed using SYBR Green Select Master Mix (ABclonal). Using β-actin as the internal standard, the data were normalized to the relative expression levels of target genes using 2^−ΔΔCT^ method. The specific primers of qPCR are shown in Table S4. Each result was biologically replicated at least three times.

### Western blot

The cells of different treatment groups were collected and lysed with radio immunoprecipitation assay buffer containing proteasome inhibitor (PL026) and phosphatase inhibitor (PL012-1). The total protein was quantified by bicinchoninic acid protein assay (Pierce). The same amount of protein was separated using SDS-PAGE, and then transferred it to polyvinylidene fluoride membranes (Millipore Corp). The polyvinylidene fluoride was sealed with 5% skim milk powder (BioFroxx, 1172GR500) for 2 h at room temperature and incubated with primary antibodies overnight at 4 °C. Then, the membranes were washed three times for 5 min each time with triethanolamine buffered saline tween buffer, followed by the second antibody incubation for 1 h at room temperature. After washing with triethanolamine buffered saline tween buffer, using enhanced chemiluminescence kit (GE Healthcare) to visualize the protein bands and analyzing with Image J software (https://imagej.net/software/imagej/).

### Coimmunoprecipitation assay

A 10-cm cell plate filled with cells, using Lipo8000 transfection reagent (Beyotime, C0533) transfect specific plasmids into cells for 48 h. Cells were collected and treated with lysate at 4 °C for 30 min and then centrifugal collection of supernatant. The supernatant was incubated with protein A/G-agarose (SantaCruz) overnight at 4 °C. After washing with sterile precooling PBS, the analysis was performed by Western blot.

### Immunoprecipitation-mass spectrometry

The cells were cultured in a 10-cm plate, using Lipo8000 transfection reagent transfect pcDNA3.1-NSP4-Flag or empty vector plasmids into cells for 48 h. Then the cells were collected and treated with lysate at 4 °C for 30 min, centrifugal collection of supernatant. The supernatant was incubated with anti-Flag antibody at 4 °C for overnight. Subsequently, the protein A/G-agarose was added to the supernatant for incubated and then centrifuged to obtain precipitate. After washing with sterile precooling PBS, the analysis was performed by SDS-PAGE. Followed by Coomassie bright blue dyeing, the corresponding lanes were cut and sent to Base Cloud Biological company for mass spectrometry.

### Determination of intracellular FFAs

FFAs were measured using Nonesterified Free fatty acids assay kit (Nanjing Jiancheng Bioengineering Institute) according to the manufacturer’s instructions. In brief, Fresh cells from different treatment groups were collected; the supernatant was collected by centrifugation at 4 °C after lysis and then determined by double-reagent method. The values were further normalized to total cellular protein content with bicinchoninic acid protein kit.

### Immunofluorescence assay

Cells grown on a confocal dish were fixed with 4% paraformaldehyde (SL1830, Coolaber) for 20 min at room temperature, followed permeabilized by 0.1% Triton X-100 (T8200, Solarbio) for 15 min. Washed three times with PBS, blocked with 5% bovine serum albumin for 1 h, and then incubated with primary antibody overnight at 4 °C. After the cells were washed with PBS for three times and incubated with fluorescent secondary antibody for 1 h. Finally, the nucleus was labeled with 4′,6-diamidino-2-phenylindole (BL105A, Biosharp) for 5 min. Cells were observed using a Leica TCS Sp8 microscope.

### LD staining

The LD was stained by BODIPY 493/503 neutral lipid drop fluorescent probe. In short, the cells were cleaned three times with PBS after fixed and permeated. The BODIPY was prepared into a 2 μM working solution and incubated at room temperature for 15 min. Digital images were obtained using the Leica TCS Sp8 microscope.

### MTT assay

MTT tests were performed using cell proliferation kits to determine the effects of the inhibitors used on cell activity and proliferation, according to the manufacturer's instructions. When 90% of Marc-145 cells were full in 96-well plates, the appropriate concentration of inhibitor was added to each well and cultured for 24 h. Then, 20 μl MTT-labeled reagents were added to each well and incubated at 37 °C for 4 h. After adding 150 μl solubilizing solution, the absorption value at 550 nm was measured by ELISA.

### Statistical analysis

Each result was independently replicated at least three times and the data were expressed as means ± SDs. All statistical were performed with Student’s *t* test (two-sided) of Prism 8 (GraphPad Software, https://www.graphpad.com/) to examine significant differences between different groups. Statistically significant differences were accepted at *p* values, ∗, *p* < 0.05; ∗∗, *p* < 0.01; and ∗∗∗, *p* < 0.001.

## Data availability

All data are included in the article and in the supplemental material.

## Supporting information

This article contains supporting information.

## Conflict of interest

The authors declare that they have no conflicts of interest with the contents of this article.
