# Supplementary material for: ACADM inhibits AMPK activation to modulate PEDV-induced lipophagy and β-oxidation for impairing viral replication
Source: J Biol Chem. 2024 Jul 11;300(8):107549. doi: 10.1016/j.jbc.2024.107549 (PMC11342783; doi:10.1016/j.jbc.2024.107549)
Supplement: Supporting Figures [file mmc1.docx]

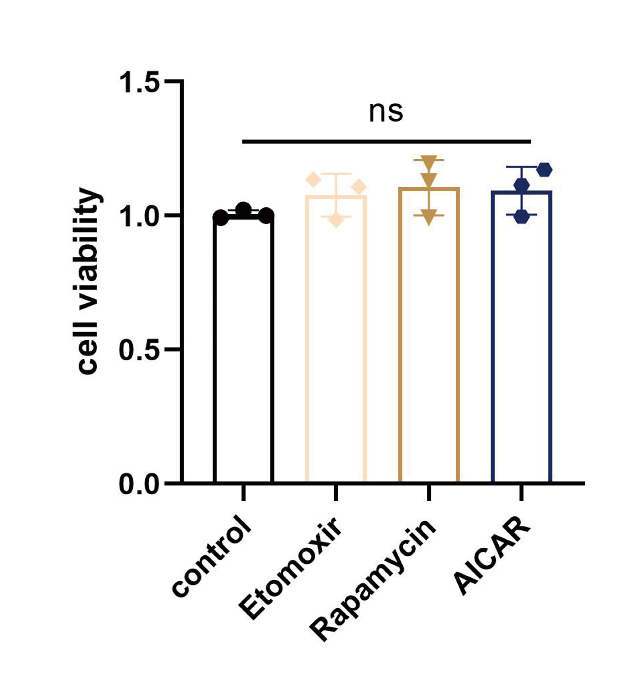
**Fig. S1 The analysis of inhibitors had no toxic effects on Marc-145 cells.** When 90% of Marc-145 cells were full in 96-well plates, the appropriate concentration of inhibitors (200μM Etomoxir; 5μM Rapamycin; 200μM AICAR) were added to each well and cultured for 24 h to performed MTT assays. The data represent three independent experiments and the difference was measured by Student’s t test, *, *P* < 0.05; **, *P* < 0.01; ***, *P* < 0.001.


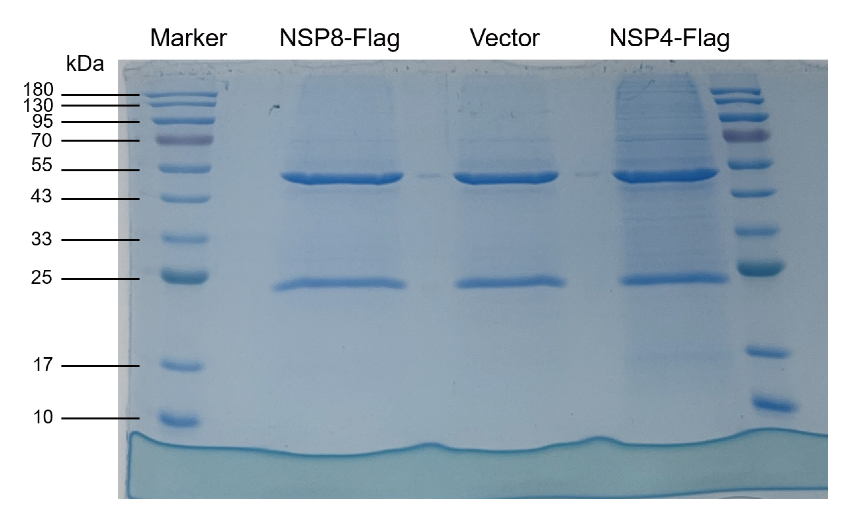
**Fig. S2 The proteins of NSP4-Flag bound were isolated by SDS-PAGE electrophoresis.** The pcDNA3.1-NSP4-Flag and vector were transfected into Marc-145 cells respectively, then anti-Flag antibody was used for immunoprecipitation to pull-down the cell proteins that may interact with PEDV NSP4. The protein samples were subjected to SDS-PAGE electrophoresis and stained with coomassie bright blue.
