# Supplementary material for: ACADM inhibits AMPK activation to modulate PEDV-induced lipophagy and β-oxidation for impairing viral replication
Source: J Biol Chem. 2024 Jul 11;300(8):107549. doi: 10.1016/j.jbc.2024.107549 (PMC11342783; doi:10.1016/j.jbc.2024.107549)
Supplement: Tables S3 and S4 [file mmc4.docx]

**Table S3 Sequences of primers used for plasmid construction in this study**

| **Purpose** | **Name** | **Sequence (5’- 3’)** |
| --- | --- | --- |
| PCR primers | NSP4-F | CGGGATCCGCCACCATGGCAGGTCTTCCTAGTTTTTC |
|  | NSP4-R | GCTCTAGACTGTAGAGTTGAATTGTAACTCAC |
|  | ACADM-F | CCAAGCTTGCCACCATGGCAGCGGGTTTCGGG |
|  | ACADM-R | CGGGATCCCGATTTTTATACTTGCCAATGTGTTC |
|  | ACADM-ΔC-F | CCAAGCTTGCCACCATGGCAGCGGGTTTCGGG |
|  | ACADM-ΔC-R | CGGGATCCCGGTCACCAATTAAAACATTTTCTTTAG |
|  | ACADM-ΔN-F | CCAAGCTTGCCACCATGGAGGAGCCGTTGATGTGTG |
|  | ACADM-ΔN-R | CGGGATCCCGATTTTTATACTTGCCAATGTGTTC |
|  | ACADM-ΔC+ΔN-F | CCAAGCTTGCCACCATGGCTTACTGTGTAACCGAAC |
|  | ACADM-ΔC+ΔN-R | CGGGATCCCGCACATCTTCGAAGACAATTCC |

Underlined sequences indicate restriction enzyme sites added for cloning

**Table S****4 Sequences of primers and siRNAs used for qPCR in this study**

| **Purpose** | **Name** | **Sequence (5’- 3’)** |
| --- | --- | --- |
| Real-time PCR primers | ACADM-F | CCGTTGATGTGTGCTTACT |
|  | ACADM-R | GCTTTTCCTCCATTGGTTA |
|  | CPT1A-F | ATGTACGCCAAGATCGACCC |
|  | CPT1A-R | TGACCACGTTCTTCGTCTGG |
|  | PPARα-F | ATCGCCCTGGCCTTCTAAAC |
|  | PPARα-R | AGATATCGTCCGGGTGGTTG |
|  | PEDV-N-F | AGATCGCCAGTTTAGCACCA |
|  | PEDV-N-R | GGCAAACCCACATCATCGT |
|  | β-actin-F | CTTAGTTGCGTTACACCCTTTC |
|  | β-actin-R | TGTCACCTTCACCGTTCCA |
| siRNA sequences | si-ACADM-F | GCUCUGAUGUAGCUGGUAUTT |
|  | si-ACADM-R | AUACCAGCUACAUCAGAGCTT |
|  | si-NC-F | UUCUCCGAACGUGUCACGUdTdT |
|  | si-NC-R | ACGUGACACGUUCGGAGAAdTdT |
